# Liquid biopsies in epilepsy: biomarkers for etiology, diagnosis, prognosis, and therapeutics

**DOI:** 10.1007/s13577-021-00624-x

**Published:** 2021-10-25

**Authors:** Jordan H. Whitlock, Tabea M. Soelter, Avery S. Williams, Andrew A. Hardigan, Brittany N. Lasseigne

**Affiliations:** 1 Cell, Developmental and Integrative Biology, The University of Alabama at Birmingham, Birmingham, AL USA; 2 Department of Neurosurgery, Duke University Medical Center, Durham, NC USA

**Keywords:** Liquid biopsy, Epilepsy, Circulating biomarkers, Nucleic acids, Cell-free

## Abstract

Epilepsy is one of the most common diseases of the central nervous system, impacting nearly 50 million people around the world. Heterogeneous in nature, epilepsy presents in children and adults alike. Currently, surgery is one treatment approach that can completely cure epilepsy. However, not all individuals are eligible for surgical procedures or have successful outcomes. In addition to surgical approaches, antiepileptic drugs (AEDs) have also allowed individuals with epilepsy to achieve freedom from seizures. Others have found treatment through nonpharmacologic approaches such as vagus nerve stimulation, or responsive neurostimulation. Difficulty in accessing samples of human brain tissue along with advances in sequencing technology have driven researchers to investigate sampling liquid biopsies in blood, serum, plasma, and cerebrospinal fluid within the context of epilepsy. Liquid biopsies provide minimal or non-invasive sample collection approaches and can be assayed relatively easily across multiple time points, unlike tissue-based sampling. Various efforts have investigated circulating nucleic acids from these samples including microRNAs, cell-free DNA, transfer RNAs, and long non-coding RNAs. Here, we review nucleic acid-based liquid biopsies in epilepsy to improve understanding of etiology, diagnosis, prediction, and therapeutic monitoring.

## Background

Epilepsy is in the top five most prevalent brain diseases alongside multiple sclerosis, stroke, Parkinson’s disease, and Alzheimer’s disease [1]. Furthermore, the condition is estimated to affect 50 million individuals worldwide, making it the most widespread chronic disease of the central nervous system [2]. Similar to diseases like cancer, epilepsy is a conglomeration of diseases and syndromes of heterogeneous etiology. Over half of all epilepsies have identifiable genetic origins; however, perturbations in brain structure, metabolic processes, and immune function can also contribute to onset [1, 3–6]. Regardless of underlying etiology, the ILAE (International League Against Epilepsy) diagnostic criteria identify persons with epilepsy by the presence of two unprovoked seizures occurring greater than 24 h apart, or a single unprovoked seizure accompanied by additional factors that contribute to a lower seizure threshold and, therefore, increased recurrence risk for a second [7]. Clinical manifestations and management vary depending on the subtype of epilepsy, ranging from relatively benign childhood diseases such as absence seizures (a brief, sudden lapse in consciousness) to severe syndromic cases like Ohtahara syndrome or focal adult-onset epilepsy [3, 4, 8, 9].

While the diagnosis of epilepsy is primarily clinical, recent technological advancements including microarrays and next-generation sequencing have become the current gold standard for understanding epilepsy etiology [10–12]. For example, Dravet syndrome is identified by *SCN1A* mutations coupled with behavioral delays, hemiconvulsion, and prolonged seizure episodes. Ohtahara syndrome, another hallmark epilepsy syndrome, is characterized by mutations in the *STXBP1* or *ARX* genes and an abnormal electroencephalogram (EEG) with a burst suppression pattern (alternating periods of inactivity and high-voltage electrical activity in the brain) [3]. Recent research has identified nearly 1000 genes associated with epilepsy [13]. A large portion of epilepsies are due to genetic factors, yet single genes only account for 1% of these cases [14]. As a result, many cases for which a genetic cause is presumed but unidentified remain. Even if a molecular or clinical diagnosis is achieved, the available treatments are limited. To date, there are multiple different approaches that result in freedom from seizures for persons with epilepsy ranging from surgery, antiepileptic drug (AED) use, and nonpharmacologic methods [15]. Surgery is usually reserved for cases of focal epilepsy, such as mesial temporal lobe epilepsy, who have failed prior medical therapy [16–19]. This leaves the remainder of epilepsy patients to pursue cocktails of AEDs, which have success in relieving 60–75% of individuals from further seizure episodes [20–22]. In addition to therapeutics, nonpharmacologic cures exist such as deep brain stimulation or dietary modifications [16, 17, 23, 24]. Due to the genetic nature of epilepsy, there is a significant need for more well-defined differential diagnostic criteria, which could enable more targeted treatment options. Thus, whether in therapeutics or diagnostics, epileptic syndromes are an area where liquid biopsies have the potential to make a substantial impact.

## Main text

### Challenges

Efforts focused on identifying the clinical utility of biomarkers in epilepsy have been undertaken for years. Compared to other diseases such as cancer, the identification, implementation, and utilization of liquid biopsies in epilepsy is underdeveloped [25–27]. The field of epilepsy presents with an abundance of phenotypic, genetic, and electrical evidence for seizures, but lacks resolution at the analyte level. This is at least partially due to various challenges stemming from epilepsy’s variable nature, availability of samples, limited cohorts, and appropriate animal models. For example, low numbers of participants underpower clinical studies [18, 28]. Additionally, the clinically variable nature of epilepsy across subtypes—most likely a result of different cofactors such as genetics, structural injuries in the brain, exposomes, microbiomes, sex, ethnicity, and age—confounds the question of the specificity and sensitivity of biomarkers in the field [5, 18, 28].

Cerebrospinal fluid (CSF), serum, plasma, and blood are among the most common liquid biopsy sample sources, yet each sample type poses unique difficulties which need to be considered. Plasma contains cellular components from lysed or apoptotic cells or anticoagulants that inhibit downstream methodologies, whereas serum does not [29]. Clotting in whole blood samples can negatively impact the availability of miRNAs and the extraction of other nucleic acids [30]. Researchers also identified a group of miRNAs (microRNAs) from platelets, suggesting that coagulation can potentially influence the miRNA profile in blood [31]. Some studies have shown a higher concentration of miRNAs, a frequently used biomarker, in serum than plasma from the same individual [31]. There have also been noted instances where different miRNA species in plasma and serum have masked other small RNA species that could contribute to biomarker profiles [32–35]. While this work has been done in cancer and neurodegenerative diseases, it could also apply to epilepsy.

In addition to sample considerations, there is a need for standardized sampling and processing techniques with respect to liquid profiling of nucleic acids [36, 37]. Fortunately, the World Health Organization specifies collection standards for blood and CSF [38, 39]. Despite this, pre-analyte processing practices still vary between individuals and clinical sites. For example, a survey of 50 randomly selected papers from 2015 reporting serum and plasma cfDNA research revealed that one-third of studies were missing methodologies for plasma and serum extraction. Furthermore, 18 articles gave no information on how cfDNA was quantified [40].

Aside from sample type and processing, the blood–brain barrier (BBB) may contribute to differences in biomarkers between CSF and blood samples [41]. The BBB is a restrictive barrier involving the microvasculature of the central nervous system (CNS). It controls the movement of cells, ions, and different molecules between the blood and the brain affecting biomarkers’ ability to enter the bloodstream thus making the identification of a universal biomarker all the more challenging [42]. Furthermore, CSF collection usually requires a lumbar puncture—an invasive procedure that involves the insertion of a large needle into the subarachnoid space of the spine [43]. Additionally, the invasive and possibly harmful nature of brain biopsy or tissue sample collection limits cohort size, and variations in technique for obtaining and/or processing samples negatively influence sample quality, therefore presenting major challenges for studies investigating the use of liquid biopsy for epilepsy.

Though the majority of epilepsy is childhood-onset and distinctly different in clinical manifestation from adult-onset epileptic disease, most biomarker studies are only performed in adults [28]. In addition, the time point during which a sample is obtained can have an effect—pre-, during, and post-seizure. A large portion of current studies obtain samples at single time points, typically post-seizure [28, 30]. Sampling pre- and post-seizure would give investigators a baseline profile to compare changes in biomarker prevalence. As prior research has shown, epileptogenesis occurs at different stages of life and can be driven by different causal factors. Single time point sample collections are restrictive and may miss crucial biomarkers in early or advanced stages of epileptogenesis as well as unintentionally capture analytes related to brain injury or other causes and not epilepsy itself [5, 30]. A previous study showed the protein expression transcriptome differs throughout time points in epileptogenesis and across seizure subtypes [44]. Biosamples can also be influenced by anti-seizure treatments, which alter the expression profile and analytes obtained from a liquid biopsy [30, 44, 45]. Outside of sample type and time of collection, there are also limitations in accurately modeling epilepsy to mirror similar onset, progression, frequency, and phenotype to that of human epilepsies [46]. Research models of pilocarpine-induced epilepsy in-vivo have shown that development and progression can occur much faster in animals compared to human models [28, 47]. Liquid biopsies may be able to address these challenges by providing more specific, quantifiable, and interpretable analytes.

### Nucleic acids (cfDNA & miRNA, and tRNAs)

Blood, including serum and plasma, and CSF liquid biopsies have been used to study the role of circulating nucleic acids such as miRNAs, transfer RNAs (tRNAs), and cell-free DNA (cfDNA) to improve diagnosis, biomarker development, and therapeutic response. These nucleic acids are often contained within exosomes released into the bloodstream from blood cells, the brain, and the spine [48]. Advances in sequencing technology have allowed higher level molecular profiling of blood, CSF, and more recently urine and saliva, to investigate nucleic acids within epilepsy outside of the exosome [49–51]. While evidence on the utility of circulating nucleic acids in epilepsy is underdeveloped in comparison to other diseases, we review the current landscape below.

### miRNAs and epilepsy

The field has not yet identified a commonly dysregulated miRNA derived from liquid biopsy, found not just within human subjects but within experimental animal models across different species as well [52]. Additionally, miRNA levels are modulated by the level of epileptogenic activity, where activity correlates with increased levels [53]. Furthermore, miRNAs play a critical regulatory role in targeting genes contributing to epileptogenesis (reviewed in [54]). miRNAs have been shown to support differential diagnosis of status epilepticus and temporal lobe epilepsy (TLE). Studies of CSF in patients with TLE and status epilepticus with matched controls revealed differential expression of 20 miRNAs [55]. However, clinical diagnosis of temporal lobe epilepsy lacked blood-based molecular biomarkers until a study of circulating miRNAs in the blood revealed altered levels of miR-27a-3p, miR-328-3p, and miR-654-3p in both humans and mice. MiR-328-3p in particular was increased in patient samples collected post-seizure [30]. Additional in situ hybridization and bioinformatic analysis in the same study showed that miR-328-3p and miR-27a-3p localized to neurons and are associated with growth factors indicated in signaling and apoptosis [30].

Pilocarpine-induced mouse models of TLE have also been used to identify differential regulation of miRNAs. 24 h post-status epilepticus, researchers sampled both blood and brain from pilocarpine models and matched controls (*N* = 16). They found significant downregulation of miR434-3p (3.8096 and 4.5716 log fold change) and miR-133a-3p (2.4585 and 2.7010 log fold change) in blood and hippocampal brain tissue, respectively [56]. Another investigation of TLE, comparing across species, revealed decreased levels of miR-219 in both kainic-acid-induced epilepsy mouse models and human epilepsy patients’ CSF [57, 58]. One pediatric study compared miRNA profiles of plasma in an assortment of focalized, general, and idiopathic epilepsy patients (*N* = 30) and controls (*N* = 20) and noted up-regulation of miR146a and miR106b (14.65-fold and 11.6-fold up-regulation respectively) in the epileptic participants [59]. Another study comparing rodents’ post-status epilepticus to patients in plasma and CSF quantified an increase in miR-134 post-seizure [60]. Based on these studies, dysregulated miRNAs could be used clinically as a diagnostic readout to reflect structural or chemical changes in the brain resulting from seizures, aiding in diagnosis or prediction. Outside of liquid sampling, multiple studies investigating miRNA regulatory roles in targeting genes have been performed in rat and mouse models [54]. In particular, miR-211 and miR-128 are involved in neuronal differentiation and branching and neuronal proliferation and excitability, respectively [54]. Identifying these miRNAs or others in liquid samples as well, would be of particular value to the field. Liquid sampling of miRNA biomarkers specifically tied to epileptogenesis in human tissues or mouse models would allow fast, non-invasive diagnosis and prognosis of epilepsy.

Outside of improving diagnostics, miRNAs have also been used to track the efficacy of treatment, specifically in identifying those who will likely be responsive or resistant to treatment. Epilepsy resolution has specific criteria: an individual must be older than the range for age-dependent epilepsies, have had no seizure activity for at least 10 years, and have gone without seizure medication for the last 5 years [7]. The majority of individuals meeting these criteria fall within the beginning stages of their adult years at the earliest. A wide range of epilepsy patients, at varying percentages across age groups, fail to respond to both medical and even surgical methods for epilepsy treatment. Moreover, a predictor for treatment responsiveness does not currently exist. Unfortunately, due to the variable efficacy of existing treatments, these individuals can or may succumb to premature death, psychosocial issues, physical injury, and reduced quality of life due to lack of effective treatment [1]. In an effort to aid treatment response in individuals, research groups have profiled the differential expression of circulating miRNAs in drug-resistant or drug-responsive epilepsies and found notable distinctions. For example, serum analysis of 30 drug-resistant patients and 30 drug-responsive epilepsy patients revealed that increased concentrations of hsa-miR-106b-5p were associated with drug-resistant epilepsies [61]. The same group discovered significantly decreased levels of miR-194-5p, -301a-3p, -30b-5p, -342-5p and -4446-3p in drug-resistant patient populations with miR-301a-3p as the most sensitive and specific predictor (80.5% sensitivity and 81.2% specificity) of nonresponse in refractory epilepsy [61]. Another case study identified elevated levels of miR-301-ap in the plasma of a drug-resistant medial temporal lobe epilepsy patient compared to 10 controls [62]. Elevated plasma levels also mirrored increased expression profiles of miR-301a-3p in hippocampal samples from the patient post-mortem. The researchers concluded that miR-301a-3p is a potentially useful biomarker of not solely medial temporal lobe epilepsy, but also may assist in the prediction of sudden and unexpected death in epilepsy [62]. In mesial TLE with hippocampal sclerosis, a study involving serum samples from 28 patients and 11 healthy volunteers, compared individuals with good surgical prognosis and poor surgical prognosis according to the Engel surgical outcome scale. RT-PCR analysis identified miR-654-3p as a potential predictor of good surgical outcomes [63]. If similar observations are found to be associated with other types of epilepsy, miRNA profiling could become a novel gold standard predictor of drug responsiveness or surgical outcomes for epilepsy procedures.

### tRNAs and epilepsy

Other recent work has researched tRNAs as a potential biomarker for epilepsy prediction, diagnosis, and monitoring. During periods of stress, tRNAs are cleaved and produce tRNA fragments (tRFs) [64]. 5'AlaTGC and 5'GluCTCtRFs have been identified by RNA-seq in plasma samples obtained from 32 focal epilepsy patients and equivalently matched controls. The study revealed varied tRFs that were elevated in patient samples pre-seizure compared to post-seizure. Participant history confirms these observations were not confounded by medications or observed in individuals with psychogenic non-epileptogenic seizures, further supporting tRFs as a potential predictor of epilepsy [64]. A follow-up study identified a third tRF, 5'GlyGCC, using electrochemical detection methods and qPCR on patient whole blood [65]. Compared to other RNA species, investigations into the role tRFs play in the diagnosis and prognosis of epilepsy are less abundant. However, the small number of studies that have been published suggest additional investigations of tRFs in pre- and post-seizure blood sampling could verify tRFs as a less invasive biomarker for predicting epilepsy risk and onset prior to seizure occurrence.

### lncRNAs and epilepsy

Similar to miRNAs and tRNAs, researchers have also studied long non-coding RNAs (lncRNAs) in the context of epilepsy. Quantification and differential expression of lncRNAs have been investigated in human tissue and animal models for mesial TLE, TLE, and Dravet Syndrome [66–70]. Like other biomarkers, lncRNAs have specific temporal and spatial patterns within the brain [71]. The use of circulating and liquid biosamples to quantify and compare lncRNAs in blood, plasma, and urine could add valuable insights into the molecular profiles of epilepsy, thus increasing understanding of epilepsy. An additional study focused on four specific lncRNAs in peripheral blood of patients versus controls (*N* = 40 per group)—HOXA-AS2, SPRY4-IT1, MEG3, and LINC-ROR. Two out of the four lncRNAs, HOXA-AS2 (Posterior beta = 1.982, *P* = 0.001) and SPRY4-IT1 (Posterior beta = 1.27, *P* = 0.02), had increased expression when comparing between patient and control groups [72]. More research is necessary to interpret the use of lncRNAs as an epilepsy biomarker.

Other studies have looked at human peripheral blood from TLE patients, comparing lncRNA ILF3-AS1 levels between serum and hippocampal tissues [73]. ILF3-AS1 is known to potentiate inflammatory cytokines and TNF-alpha expression along with increased matrix metalloproteinases, all of which are associated with epilepsy. Serum revealed elevated levels in contrast to the brain tissue, suggesting lncRNAs utility to monitor the development of TLE [73]. The remainder of lncRNA research in epilepsy is in tissues from brain and animal models, not liquid samples [68, 69, 71, 74]. lncRNAs in epilepsy have been investigated in tissues of pilocarpine and kainic acid-induced rats, wister audiogenic rat (WAR) models, lncRNA-null mice, natural antisense lncRNA mice, knockout mouse models, as well as post-mortem human brain samples [74]. However, these lncRNA findings from solid tissues could be used to validate or prioritize lncRNAs identified from liquid samples to gain additional insight and confidence in their utility as a liquid biomarker. Overall, lncRNAs are easily accessed and stable in biological fluids and can be found in apoptotic bodies or exosomes, thus supporting their wide use in epilepsy and other disease settings.

### cfDNA and epilepsy

Alternatively, cfDNA may be a potential, though largely understudied, biomarker source for epilepsy. While correlations between cfDNA and abnormal brain function are described in the literature (e.g., increased cfDNA has been described in Friedreich’s ataxia, stroke, traumatic brain injury, and other chronic brain diseases), epilepsy-specific cfDNA levels have not been investigated [75]. Limited research has profiled cfDNA concentrations in focal and extratemporal lobe epilepsies, but not epilepsies as a whole [75, 76]. Studies into cfDNA in relation to focal epilepsy are primarily focused on the concentration of cfDNA in epileptic individuals. cfDNA was measured in 167 patients with focal epilepsy, 147 of whom had refractory epilepsy, and 250 healthy control individuals. More epilepsy patients (125/167; 74.8%) had increased concentrations of cfDNA with a higher median concentration in patients (0.867 g/ml) compared to controls (0.759 g/ml) (*p* < 0.001). Symptomatic etiology was associated with increased concentrations of cfDNA compared to potentially symptomatic etiology (*p* = 0.036). Their results indicated an increased concentration of cfDNA in the blood of those with symptomatic refractory epilepsy in contrast to those with epilepsy of unknown etiology. Individuals with increased concentration of cfDNA exhibited seizures more frequently compared to those with normal concentrations [75]. Similar to miRNAs, cfDNA concentrations vary between epilepsy subtypes. A baseline concentration of cfDNA was established from the serum of 51 individuals with refractory epilepsy and 250 controls. Association analysis was performed between the cfDNA concentrations across the different subtypes of epilepsy. In contrast to focal epilepsy, research has shown extratemporal lobe epilepsy associated with an average lower level of baseline cfDNA [76]. In addition to being used as a biomarker source, cfDNA also has the diagnostic potential to be used in a targeted fashion to look for DNA mutations in known epilepsy genes [77–81]. Yet, even though cfDNA has shown tremendous utility in cancer, it is still largely uninvestigated in the context of epilepsy. As a result, cfDNA is a potential future direction for liquid biopsy biomarker discovery in epilepsy.

## Conclusions

Currently, the majority of existing epilepsy liquid biomarkers are diagnostic and could further be improved upon prior to being implemented clinically. Moreover, there are still many gaps in the field spanning across prognosis, susceptibility, and monitoring outcome or therapeutic response [82]. Liquid biopsies are a low- or non-invasive method with respect to sample collection. Because of this, CSF analysis could be used in tandem to increase the diagnostic yield of next-generation sequencing by blood. Comparisons between the two liquid biosamples and solid tissues may allow researchers to draw parallels between the accuracy of using biopsy by blood as a proxy to understand which nucleic acids present in the brain and provide a less invasive sampling method if blood correlates well to CSF or findings in solid tissues from animals and human. Additionally, there may be underutilized sample sources; the urine liquid sampling space is largely untouched in the field [74].

Future research should account for the heterogeneous nature of epilepsy and the high likelihood of multiple possible etiologies for any given epilepsy [82]. Recognizing this, liquid biopsy studies are expanding to aid in the identification of somatic mutations, which may shed light on some of the unknown drivers of epilepsy [77–81]. Somatic mutations arise during development, and the exact time point they are created can influence whether a variant is widespread across multiple tissues or restricted to just one, further contributing to heterogeneity in presentation [83]. As mentioned previously, over half of all undiagnosed cases of epilepsy are due to an unknown genetic cause [3]. There is an extremely high rate of brain-only somatic mutations that have been shown to be causal [77]. Especially in unsolvable developmental and epileptic encephalopathies, mosaicism studies have been completed using blood, CSF, and saliva from participants and successfully identified causal genetics [80, 81, 83]. However, the BBB and the invasive collection nature of obtaining brain tissue samples provide a major barrier to furthering research in these areas. The percentage of epilepsies attributed to somatic mosaicism could be largely underrepresented in the field at the moment [83]. Additional research using liquid biopsies of the CSF, blood, saliva, and serum as a less invasive approach could revolutionize diagnosis and future treatment of somatic mutations in the brain.

To date, there still exist no predictive pre-seizure biomarkers to foresee the onset of an individual’s next seizure before its occurrence [84]. A combination of biomarkers, to match the heterogeneity of the disease, may push the field closer to predicting and identifying epilepsy etiology. In the same light, progression and the onset of epilepsy is highly variable—as Liimatainen said, “seizures beget seizures” [75]. Therefore, there is a significant need for serial sampling at multiple time points before, during, and after seizures in the future, instead of single time point extractions [30]. Some research using paired serum and saliva samples from persons with epilepsy has shown that saliva concentrations correlate with serum-free valproate concentration, suggesting saliva may be a proxy for therapeutic drug monitoring of valproate in the clinical setting [85]. Serial sampling could also be beneficial in studies aimed at identifying effective treatments for epilepsy. All in all, this cannot be done without better models or systems that match the human epilepsy phenotype—a challenge not just in this subfield but in all sectors of CNS research. Liquid biopsies in epilepsy will further our understanding of etiology, diagnosis, prediction, and therapeutics.

## Data Availability

Not applicable.

## Abbreviations

AEDs: Antiepileptic drugs
BBB: Blood–brain barrier
CNS: Central nervous system
cfDNA: Cell-free DNA
CSF: Cerebrospinal fluid
EEG: Electroencephalogram
ILAE: International League Against Epilepsy
miRNA: Micro-RNA
TLE: Temporal lobe epilepsy
tRFs: tRNA fragments
tRNA: Transfer RNA
WAR: Wister audiogenic rat
